# Saving the Children

**Published:** 1920-08-28

**Authors:** 


### Saving the Children.

Women magistrates, women jurors, and women police have come to stay. It would be folly to expect too much from the innovation. The advent of women into politics and into the scheme of administrative justice will not produce a new heaven and a new earth. Like men, they will have to learn from experience, and they will make many mistakes and suffer bitter disillusionment when they come face to face with human nature as it is revealed in the courts of law.

But we believe that, on the whole, women magistrates will have a humanising influence in the application of the penal code; that they will realise more intuitively than men the importance of the personal equation; and that, as far as the jurors are concerned, they will bring an element of imagination to bear in the trial of prisoners which nobody who has an intimate acquaintance with our criminal courts would say distinguishes the average collection of the twelve good men and true who have up to now formed the backbone of British justice. One's only adverse comment on the selection of women jurors is that the age-limit—twenty-one years to sixty-five—is far too wide, especially at the very outset of the new system. It would have been adequate and wise to fix the earlier limit at thirty —the age at which a woman is at present entitled to the vote.

The most practical way in which the influence of women can be usefully and immediately employed is in the children's courts. Public opinion is compelling the Home Office to devise more satisfactory methods of trying boys and girls for the offences of childhood, and of eliminating, as far as possible, the atmosphere of the police court and the man in blue. The association of women on the Bench with the stipendiary magistrate should have a definite advantage in adding feminine discernment to knowledge of the law, in ensuring that mentally defective or abnormal children are not punished for offences for which they cannot be held responsible, in the sympathetic investigation of family history, in freeing boys and girls of unfortunate parenthood from their evil home circumstances, and generally in extending a benevolent and watchful influence upon that child-life which, morally and physically, can be saved or finally wrecked in the children's courts.

